# Bioinspired Confined Assembly of Cellulosic Cholesteric Liquid Crystal Bubbles

**DOI:** 10.1002/advs.202308442

**Published:** 2024-01-15

**Authors:** Qiao Wang, Zhuohao Zhang, Chong Wang, Xinyuan Yang, Zhonglin Fang, Luoran Shang

**Affiliations:** ^1^ Shanghai Xuhui Central Hospital Zhongshan‐Xuhui Hospital, and the Shanghai Key Laboratory of Medical Epigenetics the International Co‐laboratory of Medical Epigenetics and Metabolism (Ministry of Science and Technology) Institutes of Biomedical Sciences Fudan University Shanghai China

**Keywords:** cholesteric liquid crystals, confined assembly, hydroxypropyl cellulose, microfluidics, solvent extraction, structure color

## Abstract

Construction of biomimetic models for structural color evolution not only gives new photonic phenomena but also provide cues for biological morphogenesis. Here, a novel confined self‐assembly method is proposed for the generation of hydroxypropyl cellulose (HPC)‐based cholesteric liquid crystals (CLCs) microbubbles. The assembly process relies on the combination of droplet microfluidics, solvent extraction, and a volume confined environment. The as‐prepared HPC structural color microbubbles have a transparent shell, an orderly arranged cholesteric liquid crystal (CLC) middle layer, and an innermost bubble core. The size of the microbubble, shell thickness, and the color of the CLC layer can be adjusted by altering the microfluidic parameters. Intriguingly, benefited from the compartmentalization effect provided by droplet microfluidics, microbubbles with multiple cores of different color combinations are generated under precise control. The self‐assembled CLCs microbubbles have bright structural color, suspending ability, and good temperature‐sensitive characteristics, making them ideal underwater sensors. The present confined assembly approach will shed light on creating novel photonic structures and the HPC microbubble will find widespread applications in multifunctional sensing, optical display, and other related fields are believed.

## Graphical abstract

HPC microbubbles are prepared by solvent extraction of microfluidic‐derived microcapsules encapsulating a low‐concentration HPC solution. The continuous water loss of the microcapsules concentrates HPC, and induces the assembly of HPC in the volume confined environment, eventually forming microbubbles with a transparent resin shell, a structural color middle layer, and an innermost bubble core.

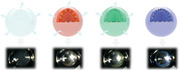

## 1 Introduction

Structural color is a widespread phenomenon in natural organisms, which is generated by the interaction between light and certain periodic physical structures.^[^ ^1^, ^2^, ^3^, ^4^ ^]^ Due to its intrinsic coloration mechanism, structural color is stable and easy‐perceivable, and these advantages have long been an impetus for the manufacture of artificial structural color materials.^[^ ^5^, ^6^, ^7^, ^8^ ^]^ Through either microfabrication methods or self‐assembly of basic units, such as colloidal nanoparticles and block copolymers, structural color materials with various configurations and functions have been constructed, showing extensive application values in anti‐counterfeiting, optical display, sensing, and biomedical devices.^[^ ^9^, ^10^, ^11^, ^12^, ^13^, ^14^, ^15^ ^]^ Although numerous efforts have been made, a large portion of them focus on creating solid photonic structures including microgrooves, stackings, three‐dimensional colloidal crystals,^[^ ^16^, ^17^, ^18^, ^19^, ^20^, ^21^ ^]^ etc. In many natural creatures, the structural coloration is formed by biomolecular composition of liquid crystal (LC) organization, a self‐assembled state with diverse ordered structures showing distinct properties. However, reproducing such organizations in artificial systems is relatively less explored.^[^ ^22^, ^23^, ^24^ ^]^ Additionally, biomolecules in vivo are often subjected to a small‐scale, volume confined environment. Thus, construction of a biomimetic model for structural color evolution via LC assembly in a confined geometry will not only give rise to new phenomenon but also provide cues for the many aspects of biological morphogenesis and behaviors.^[^ ^25^, ^26^, ^27^, ^28^ ^]^

**Figure 1:**
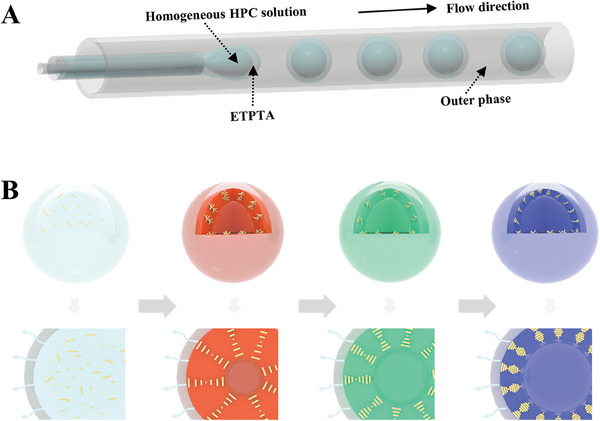
Schematic illustration of A) the generation process of the HPC microcapsules through microfluidics; B) confined self‐assembly of HPC molecules into CLC microbubbles by solvent extraction.

Herein, we propose a bioinspired confined self‐assembly strategy for the generation of cellulosic structural color, as illustrated in **Figure** **1**. The confined environment is provided by microfluidic‐derived microcapsules encapsulating low‐concentration hydroxypropyl cellulose (HPC) molecules, which are the assembly units. Droplet microfluidics is a technique to precisely manipulate and produce diverse droplets via multi‐phase immiscible fluids in microchannels. Based on its miniaturization, controllability, and high throughput, droplet microfluidics has great application values in the fields of biomaterial fabrication, biochemical reactions, biomedical analysis, etc.^[^ ^29^, ^30^, ^31^, ^32^, ^33^ ^]^ Here, the microfluidic‐derived HPC microcapsules have a transparent and semipermeable shell as well as a homogeneous core. We find that, through a simple solvent extraction process, the HPC molecules in the core become concentrated, which initiates the transition from a homogeneous phase into a cholesteric liquid crystal (CLC) phase, as manifested by the emergence of structural color. Meanwhile, a microbubble is generated in the core during the process and expands with the continuous solvent extraction, which results in blue shift of the structural color. Based on this, cellulosic microbubbles with uniform structural color are generated; by adjusting the microfluidic flow rates and initial HPC concentration, microbubbles with multiple cores of different color combinations can be obtained. In addition, the HPC microbubbles can perfectly preserve the original temperature sensing characteristics of HPC, and their suspending ability make them potential underwater optical sensors. The results demonstrate a novel bioinspired confined assembly strategy of cellulosic cholesteric liquid crystals (CLCs). We believe this study will shed light on the fabrication of structural color materials and exploration of their applications.

## 2 Results and Discussion

**Figure 2:**
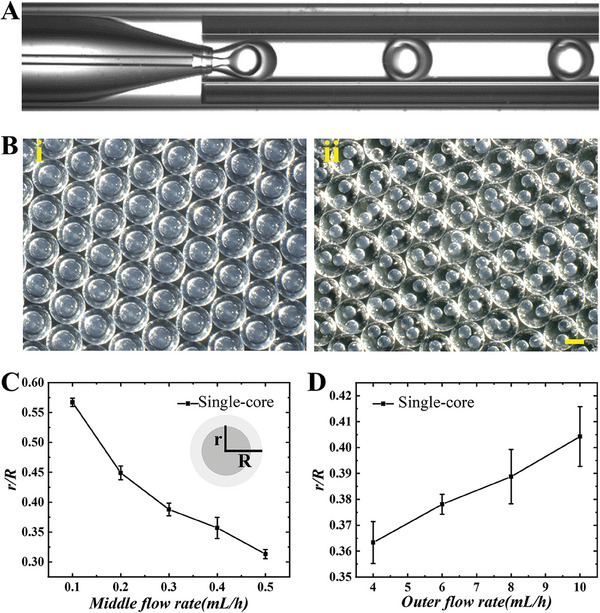
A) Real‐time images of the generation of single‐core double emulsion droplets; B) microscopic images of i) single‐core and ii) double‐core microcapsules under reflected light; (C) plot of *r*/*R* as a function of the middle phase flow rate. The internal and external flow rates were set at 30 µL h^−1^ and 5 mL h^−1^, respectively; D) plot of *r/R* as a function of the external phase flow rate. The internal and middle flow rates were fixed at 30 µL h^−1^ and 0.3 mL h^−1^, respectively. The scale bar is 200 µm.

In a typical experiment, the HPC microcapsules were first prepared by double‐emulsion microfluidics using a capillary chip consisted of coaxially aligned glass tubes (Figure S1, Supporting Information). An aqueous solution of HPC mixture served as the inner phase. Since high‐concentration HPC typically has a large viscosity, making it difficult for microfluidic processing, we here chose a low‐concentration HPC solution (in the range of 5%−28%). In addition, acrylamide (AM) was added in the inner phase to promote the later self‐assembly process, and carbon nanotubes (CNTs) were added as a dark background agent to enhance color saturation. Trimethylolpropane ethoxylate triacrylate (ETPTA) monomers together with the photoinitiator was used as the intermediate phase. An aqueous solution of surfactant Poly (ethylene glycol)‐block‐poly(propyleneglycol)‐block‐poly (ethylene glycol) diacrylate (F108) was used as the external phase. With that, water‐in‐oil‐in‐water (W/O/W) double emulsions were generated continuously, as shown in **Figure** **2**A.

Then the double emulsion droplets were cured under ultraviolet (UV) irradiation to form microcapsules with a uniform spherical shape (Figure 2B; Figure S2, Supporting Information). Additionally, by adjusting the flow rate of the internal, middle, and external phase fluids, HPC microcapsules with different core size as well as number of cores can be obtained (Figure 2B; Figure S3, Supporting Information). In particular, we investigated the effects of different flow rates on the shell thickness of the microcapsules, as this parameter is key to the status of the microcapsules subjected to subsequent solvent extraction.^[^ ^34^, ^35^ ^]^ Within a certain range of flow rates where the microcapsules had a single core, when the flow rate of the inner and outer phases were fixed, the ratio of the inner core radius (*r*) to the entire microcapsule radius (*R*) decreased with the increasing of the middle flow rate (Figure 2C). On the contrary, when the flow rates of the internal and middle phases were constant, *r*/*R* increased with the external phase flow rate (Figure 2D). These results indicated that by tuning the flow rates, microcapsules with different shell thickness can be obtained.

**Figure 3:**
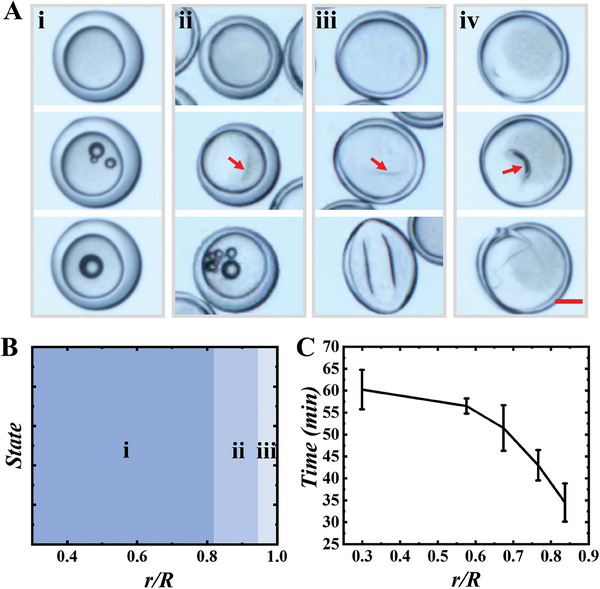
A) Microscopic images of the response state of HPC microcapsules with different shell thicknesses in ethanol; B) state diagram showing different responses of the microcapsules as a function of *r*/*R*. i) When *r*/*R* was in the range of 0.3–0.818, there was no obvious change in the microcapsule shell, and bubbles were generated in the core directly. ii) When *r*/*R* was in the range of 0.818–0.945, the shell of the microcapsules buckled, creating dimples and then generating bubbles. iii) When *r*/*R* was larger than 0.945, there were no bubbles generated, and the microcapsules eventually evolved into a bowl shape or burst in the shell; C) the time for the onset of bubble in the HPC microcapsules as a function of shell thickness. The scale bar is 100 µm.

The as‐prepared HPC microcapsule had a transparent shell, which encapsulated HPC‐AM mixture in a confined space and isolated it from the external environment, ensuring the stability of HPC properties and facilitating its self‐assembly at the later stage. Based on this, we used ethanol as a solvent extraction agent to concentrate the initial low‐concentration HPC. Due to the semipermeability of the ETPTA shell,^[^ ^36^ ^]^ water in the inner core of the microcapsules was drained out, while HPC molecules were retained. We studied the response of the HPC microcapsules with different shell thicknesses in response to the solvent extraction process. We found that HPC microcapsules with a relatively thick shell show no significant change in their shell layer during exposure to ethanol. The continuous loss of water induced a sudden emergence of bubbles in the core (**Figure** **3**Ai). In contrast, microcapsules with thinner shells initially formed a wrinkled dimple, which become deepened with time, until an abrupt bubble formation when the shells returned to its original shape (Figure 3Aii). Microcapsules with ultra‐thin shells did not show bubble formation during the whole water extraction process; their shells were sunken to the extreme and finally formed a bowl‐like structure or ruptured (Figure 3 Aiii,vi; Figure S4, Supporting Information). These different responses were summarized in a state diagram as a function of *r/R*, as shown in Figure 3B. In addition, we found that the shell thickness also affected the time for the onset of bubble generation in the microcapsules. As shown in Figure 3C, microcapsules with thinner shells had shorter time for bubble generation.

**Figure 4:**
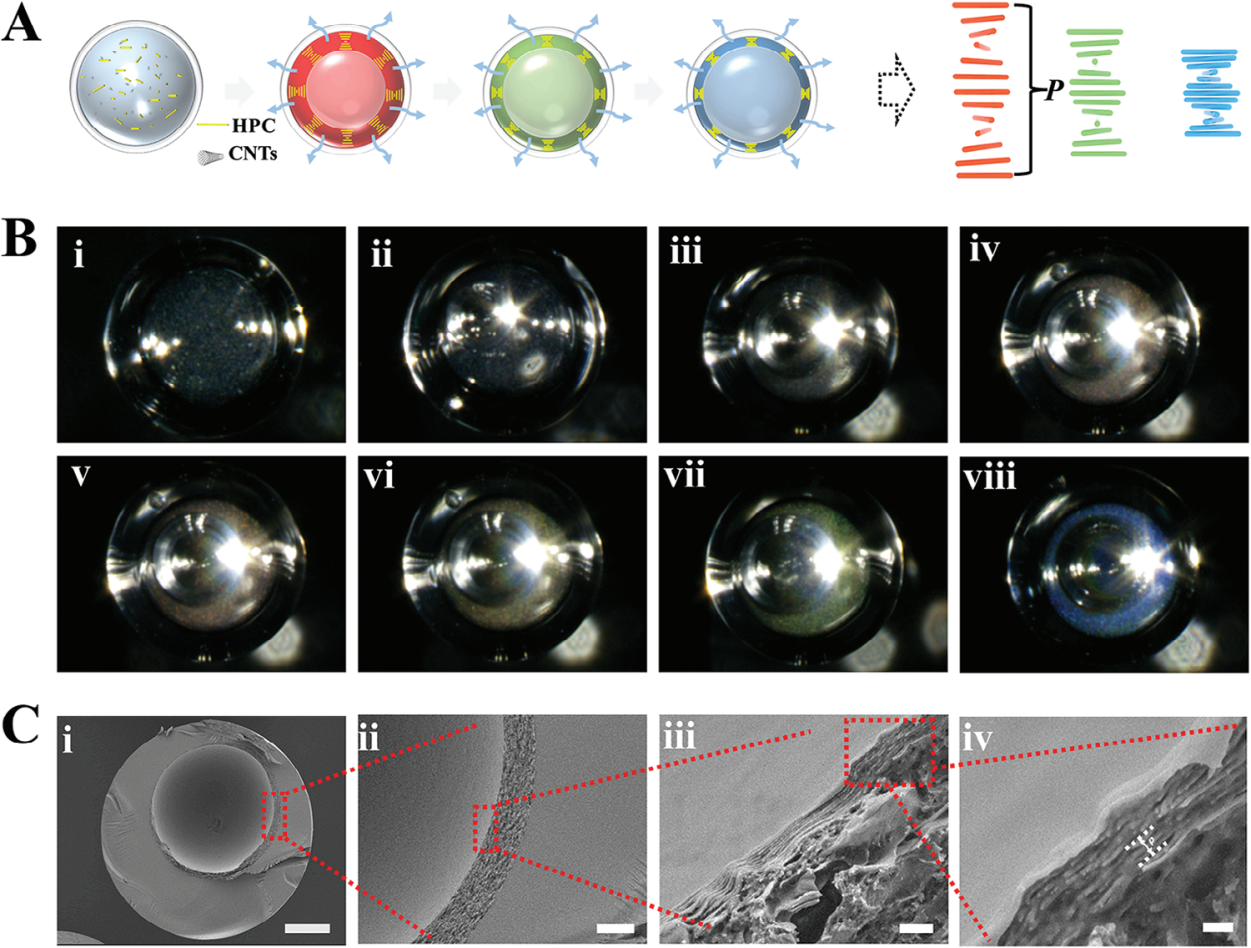
A, B) Schematic diagram and (B) real‐time microscopic images showing the confined self‐assembly process of HPC in the microcapsules by solvent extraction; C) i) cross‐sectional SEM images of a single‐core HPC microbubble; ii) magnified view showing the double‐shell structure; iii, iv) magnified view showing the ordered structure of the HPC layer. The scale bars are 100 µm in (B) and Ci), 20 µm in Cii), 1 µm in Ciii), and 300 nm in Civ), respectively.

We then investigated the confined assembly of HPC by selecting microcapsules with a relatively thick shell so that their response to the solvent extraction process was more stable and tunable. HPC is a hydrophilic cellulose derivative, which forms hydrogen bonds with water molecules through the hydroxyl groups. When its concentration reaches 50%−70%, HPC can self‐assemble into CLCs with a periodic helical structure, which generates a photonic band gap that prohibits the propagation of light within a certain range of wavelength, reflecting visible light selectively.^[^ ^37^, ^38^ ^]^ As shown in **Figure** **4**, the initial microcapsules encapsulating a low‐concentration (20%−28%) HPC mixture solution did not show any color. After immersion in ethanol, bubble emerged within the microcapsules and grew over time. Then, at a certain point, structural color appeared, indicating a homogeneous‐to‐CLC phase transition of HPC with the increase of concentration. We used a scanning electron microscope (SEM) to characterize the microstructure features of the completely dried HPC microbubbles. As shown in Figure 4C, the microbubbles had a clear‐defined double‐shell capsule‐like structure with an outer shell of ETPTA resin and inner shell of HPC CLCs, which exhibited an ordered structure (Figure 4Civ). The reflected light wavelength of CLCs is determined by the helical pitch (*p*), which is the distance between two molecular layers with 360° twisting. Thus, during the process of solvent extraction, the concentration of HPC gradually increased, resulting in a decrease of *p* value and a corresponding blue shift of the structural color (Figure 4A,B). Over the entire process of solvent extraction, the microcapsules appeared red, green, and blue gradually, together with the expansion of the bubble. Interestingly, the water drain‐out rate affects the speed of the bubble HPC layer assembly, so the color of the microbubbles at a certain time point can be manipulated by controlling the water drain‐out rate (Figure S5, Supporting Information). In addition, the transparent shell layer of microbubbles showed rather strong reflection, and we found that decreasing the thickness of the microbubble shell (on condition that *r*/*R* was in the range where bubble generation and CLC assembly can happen) can improve a bit of the color appearance of the intermediate layer (Figure S6, Supporting Information).

**Figure 5:**
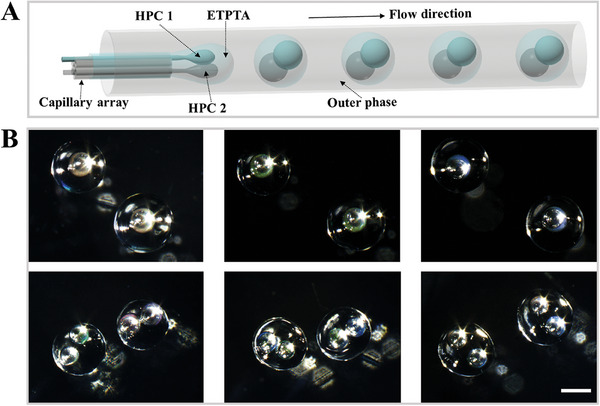
A) Schematic diagram of the preparation of multicore microcapsules encapsulating HPC mixtures of different initial concentrations through a capillary array‐based microfluidic device; B) microscopic images of the single‐core and double‐core HPC CLCs microbubbles with different colors and color combinations. The scale bar is 200 µm.

Based on this confined assembly strategy, we generated HPC microbubbles with diverse colors by leveraging the flexible fluid controllability of microfluidics. We constructed a double emulsion microfluidic chip using a seven‐bore capillary array as the injection tube (Figure S7, Supporting Information). Different inner fluids can be injected simultaneously via separate bores, thus obtaining multi‐core microcapsules. As depicted in **Figure** **5**A, two flow of HPC mixture solutions with different initial concentrations (HPC1 and HPC2) were injected into two bores of the injection tube at the same time, and double‐core microcapsules were obtained. In addition, during the subsequent solvent extraction process, the self‐assembly of HPC in each core occurred independently without mutual interference. This was enabled by the intricate design of the multi‐core capsule structure after solidification of ETPTA, which provided compartmentalized and confined spaces for assembly. Due to the difference in the initial HPC concentration in different cores, their *p* value was different at the same time point of the solvent extraction process. The resultant HPC microbubbles thus presented different structural colors, as shown in Figure 5B. Moreover, by injecting three inner flows through the capillary array injection tube, HPC microbubble with three different color combinations were fabricated (Figure S8, Supporting Information).

**Figure 6:**
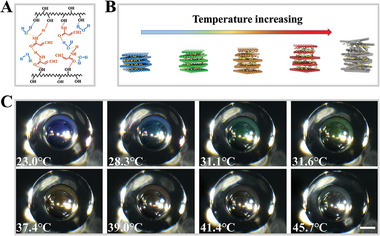
A) Schematic diagram of the hydrogen bonds between HPC, AM, and water molecules; B) schematic illustration of the change in *p* during heating of AM‐doped HPC mesophase; C) microscopic images of the temperature sensing process of the AM‐doped HPC microbubbles. The scale bar is 100 µm.

Temperature sensitivity is an inherent property of HPC mesophase. Typically, at higher temperatures, the structural color of HPC CLCs red‐shifts due to increase of *p*.^[^ ^39^, ^40^, ^41^ ^]^ We reasoned that the as‐prepared HPC microbubbles would retain this property since the ETPTA resin shell provided a stable and transparent shield. In addition, AM molecules can form hydrogen bonds with both HPC and water molecules (**Figure** **6**A), which facilitates the self‐assembly of HPC and does not impede the intrinsic temperature‐responsiveness of HPC (Figure 6B).^[^ ^42^ ^]^ To validate this, the HPC microbubbles were placed in a dish and heated by water bath. As shown in Figure 6C, the color of the HPC microbubbles red‐shifted with increase of the temperature. Because the microbubbles were submerged in water, the HPC mesophase in the core of the HPC microbubbles did not suffer from much water loss when heated at a modest temperature range. Thus, such color shift was attributed to the thermotropic feature of HPC with a positive temperature dependence of *p*. The color of the initially blue HPC microbubbles displayed a red shift in the temperature range of 23.0–45.7 °C, and it vanished above 45.7 °C. Considering that the geometric features of the HPC microbubbles can be finely adjusted, their overall density can be controlled to match a given solution, and thus fully suspended to serve as flexible temperature sensors. Likewise, the mechanical sensing properties of HPC are demonstrated to be maintained in microbubbles, making it potentially useful in the field of mechanical sensing as well (Movie S1, Supporting Information).

## 3 Conclusion

In summary, we have proposed a confined environment to let low‐concentration HPC self‐assemble into CLCs microbubbles. The method combined the use of microfluidics and a solvent extraction process. Initially, microcapsules encapsulating a low‐concentration HPC‐AM mixture were prepared via droplet microfluidics. Then the as‐microcapsule were placed in ethanol for solvent extraction, which resulted in HPC molecules being concentrated and self‐assembled into an ordered periodic structure, forming HPC CLCs microbubbles. The prepared HPC CLCs microbubbles had a transparent shell layer, a structural color middle layer, and an internal bubble core. By adjusting the microfluidic parameters, the shell thickness was finely tuned such that the assembly process of HPC could be regulated. Additionally, by employing microfluidic devices with a capillary injection tube, microbubbles with multiple cores of different color combinations were formed. Moreover, the microbubbles displayed excellent optical sensor abilities benefited from their maintenance of the intrinsic temperature responsive feature of HPC. These properties make the as‐prepared microbubbles have great potential applications in multiple fields, such as anti‐counterfeiting, information encryption, temperature detection and biosensors.

In general, the present LC self‐assembly method is simple and easy to control. However, there are some limitations. For example, the reflection of the microbubble shell interferes the measurement of the reflection spectra of the CLC layer, which needs to be resolved in our future work. Additionally, the seven‐bore capillary microfluidic device is currently applicable to the injection of up to three internal phase fluids, so that the injection channel of each is separated apart to prevent pre‐mixing. To further increase the number of the internal phase fluids and the cores of the microbubbles, proper wettability modification of the capillary tube or refinement of the microfluidic device may be required. Overall, we believe the confined self‐assembly method opens a promising pathway for the construction of novel structural color materials.

## 4 Experimental Section

### Materials

HPC was bought from Nippon Soda Co., Ltd. ETPTA (average Mn ≈428), F108(average Mn ≈14600), and 2‐hydroxy‐2‐methylpropiophenone (HMPP) were purchased from Sigma‐Aldrich. AM, ethanol was bought from Sinopharm Chemical Reagent Co., Ltd. CNTs(dispersed solution) were obtained from XF NANO Materials Tech Co., Ltd. Deionized water was obtained from a Milli‐Q Plus 185 water purifying system (Millipore).

### Fabrication of The Microfluidic Device

The microfluidic chip was fabricated with capillary glass tubes, a glass slide, and injection needles. The inner tube was a single‐bore capillary or seven‐bore capillary array, and was tapered by a portable Bunsen burner and polished on an abrasive paper. The inner tube was embedded coaxially into the middle tube, whose inner and outer diameters were 580 µm and 1 mm, and nozzle was treated by the same way as aforementioned. The middle tube was nested into the external tube, and all of these tubes were fixed to the glass slide. After that, dispensing needles were anchored at the junctions of two tubes, and epoxy resin was used to fill the interstices where necessary.

### Generation of HPC Structural Color Microbubbles

The inner phase HPC solution in the concentration range of 5%−28% was prepared. For the preparation of an AM‐doped solution containing 28% HPC, 10 mL solution containing 2.8 g HPC, 0.28 g AM (monomers), 0.01 g CNTs and the remaining deionized water were mixed in a lightproof mixer for 48 hours, then collected in a centrifuge tube and centrifuged (10000 rpm, 10 min) to remove air bubbles. The mixture was stored at 4 °C out of the light. The photocurable mixture of ETPTA and 1%HMPP was the middle phase, and the 2% wt F108 solution was the outer phase. The fluids stored in glass syringes were driven by syringe pumps to flow through the microfluidic chip via polyethylene tubes. Double emulsion droplets were formed and solidified by UV irradiation in the collection tube and HPC microcapsules were generated. The core size, number of cores, and shell thickness were adjusted by the flow rates of three phases. Multiple inner fluids with different concentrations of HPC can be simultaneously injected into the microfluidic chip with a seven‐bore capillary array injection tube. Finally, the as‐prepared microcapsules were immersed in ethanol for 6–10 h and observed under a microscope.

### Response of the HPC Microcapsules During Solvent Extraction

An AM‐mixed 5% HPC solution was used as the inner phase, a mixture of ETPTA and 1%HMPP was the middle phase, and a 2% wt F108 solution was the outer phase. Each phase solution was injected into the microfluidic chip to prepare microcapsules as mentioned above. The external phase flow rate was set at 3 mL h^−1^, the middle phase flow rate was fixed in 0.3 mL h^−1^, and the inner phase flow rate was gradually adjusted at 5, 40, 80, 120, 160, 200, 240, and up to 400 µL h^−1^. After UV irradiation, HPC microcapsules with different shell thicknesses were prepared. Then they were placed in ethanol and the different status including shell buckling, bubble formation, etc. were observed in real‐time.

### Underwater Temperature Sensing of HPC Structural Color Microbubbles

Under white light irradiation, blue AM‐HPC microbubbles placed on a holder inside a dish filled with water on a heating plate for water bath heating. The temperature of the heating plate was gradually raised (in the range 20—90 °C), each time by 3 °C. Afterward, the temperature of the aqueous solution was measured with a thermometer and the change of the structural color during the course was observed.

### Characterization

The microstructure of the HPC microbubbles was characterized by SEM(Sigma 300, ZEISS) after drying and sputter coating. Optical microscopic images of the HPC microcapsules and structural color microbubbles were observed by a stereomicroscope (SNZ818, Nanjing) and were imaged with a charge coupled device (CCD, Sony, E3ISPM).

## Conflict of interest

The authors declare no conflict of interest.

## Supporting information

Supporting Information

Supplemental Movie S1

## Data Availability

The data that support the findings of this study are available from the corresponding author upon reasonable request.;
