# Supplementary material for: Bioinspired Confined Assembly of Cellulosic Cholesteric Liquid Crystal Bubbles
Source: Adv Sci (Weinh). 2024 Jan 15;11(11):2308442. doi: 10.1002/advs.202308442 (PMC10953211; doi:10.1002/advs.202308442)
Supplement: Supplementary file 1 — Supporting Information [file ADVS-11-2308442-s002.pdf]

ADVANCED SCIENCE
Open Access

Supporting Information

for *Adv. Sci.*, DOI 10.1002/advs.202308442

Bioinspired Confined Assembly of Cellulosic Cholesteric Liquid Crystal Bubbles

*Qiao Wang, Zhuohao Zhang, Chong Wang, Xinyuan Yang, Zhonglin Fang and Luoran Shang**

Supporting Information

Bioinspired Confined Assembly of Cellulosic Cholesteric Liquid Crystal Bubbles

Qiao Wang[1], Zhuohao Zhang[1], Chong Wang[1], Xinyuan Yang[1], Zhonglin Fang [1], Luoran Shang[1]*

[1]Shanghai Xuhui Central Hospital, Zhongshan-Xuhui Hospital, and the Shanghai Key Laboratory of Medical Epigenetics, the International Co-laboratory of Medical Epigenetics and Metabolism (Ministry of Science and Technology), Institutes of Biomedical Sciences, Fudan University, Shanghai, China.

* Corresponding author

Email: luoranshang@fudan.edu.cn

1. Supporting images

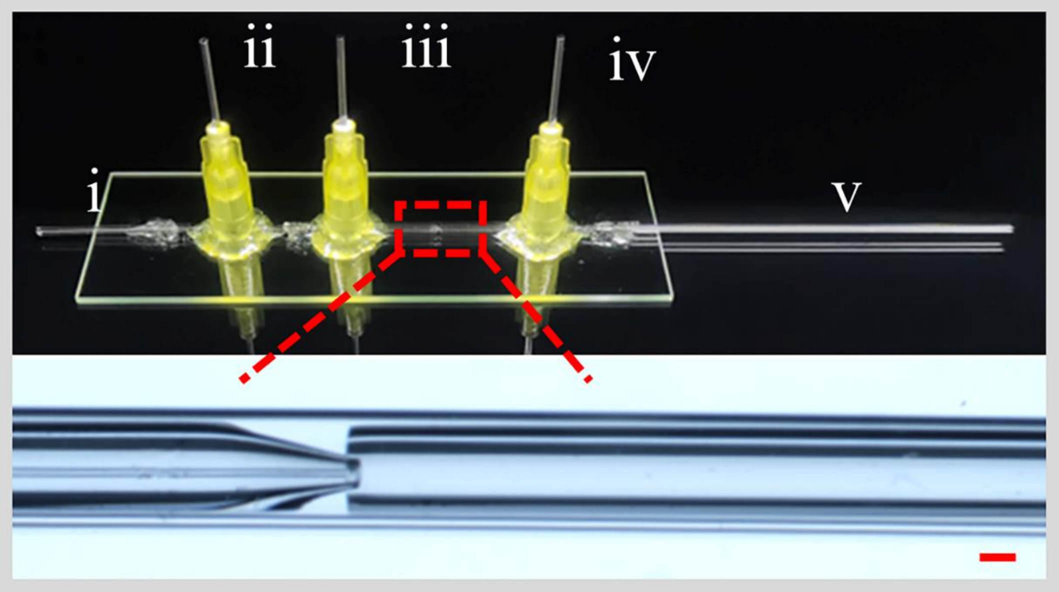

Figure S1. (Top) a photograph of the capillary microfluidic chip; i, ii, iii, iv, and v represents the inner phase injection tube, middle phase inlet, vent outlet, outer phase inlet, and collection tube,

respectively. (Bottom) a microscopic image showing the coaxially aligned geometry of the capillaries. Scale bar is 200μm.

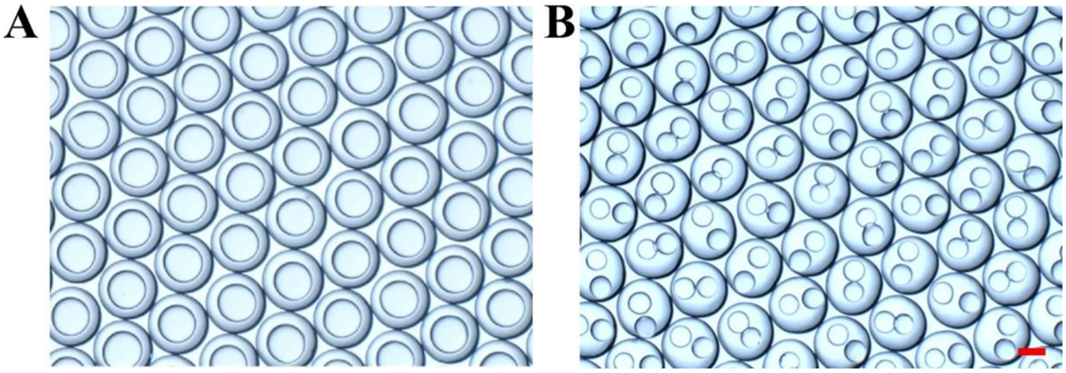

Figure S2. Microscopic images of (A) single-core and (B)double-core low-concentration HPC-encapsulated microcapsules under transmitted light, corresponding to **Figure 2B**. The scale bar is 200μm.

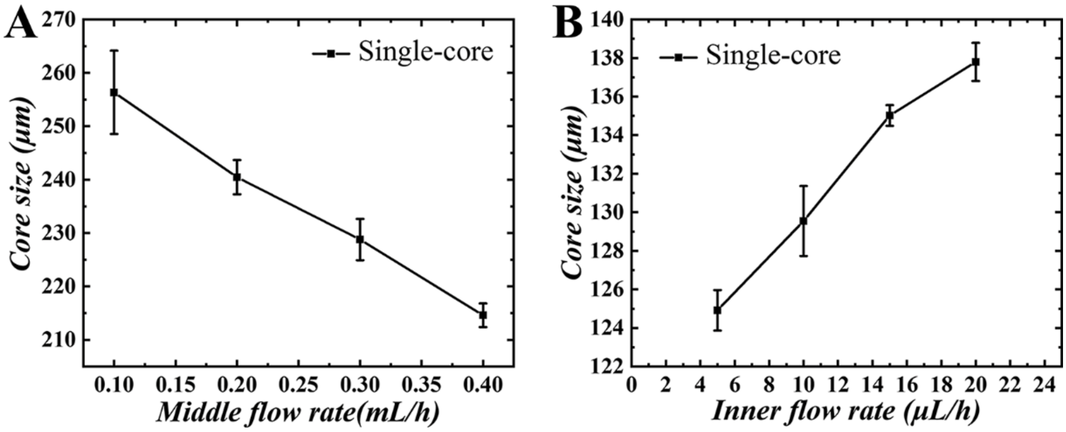

Figure S3. (A) Plot of the core size (under the single-core state) of the microcapsules as a function of the middle flow rate. The inner and outer flow rates were set at 50μL/h and 5mL/h, respectively. (B) Plot of the core size (under the single-core state) of the microcapsules as a function of the inner flow rate. The middle and outer flow rates were fixed at 0.3mL/h and 5mL/h, respectively.

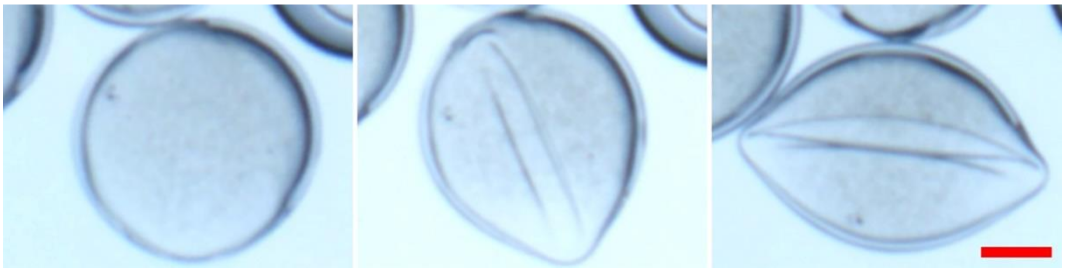

Figure S4. Microscopic images of the response of ultrathin shell microcapsules with unevenly distributed shell thickness to ethanol. The scale bar is 100μm.

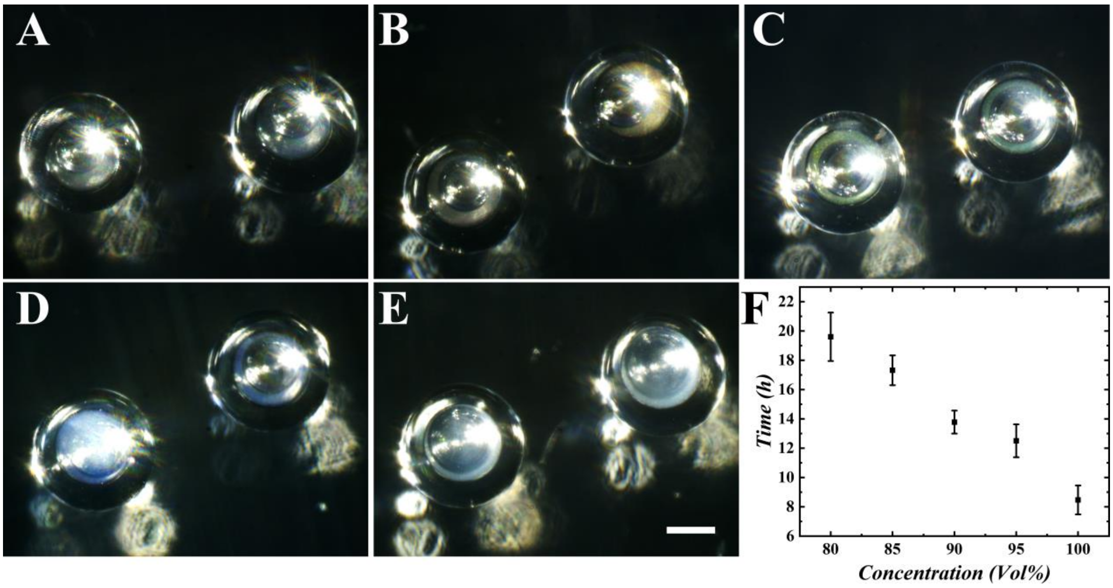

Figure S5. (A-E) Microscopic images of microbubbles after 11.16h solvent extraction at 92%, 94%, 96%, 98%, and 100% ethanol solution, respectively. (F) Plot of time for microcapsules self-

assembled into blue CLCs as a function of ethanol concentration. The scale bar is 200μm.

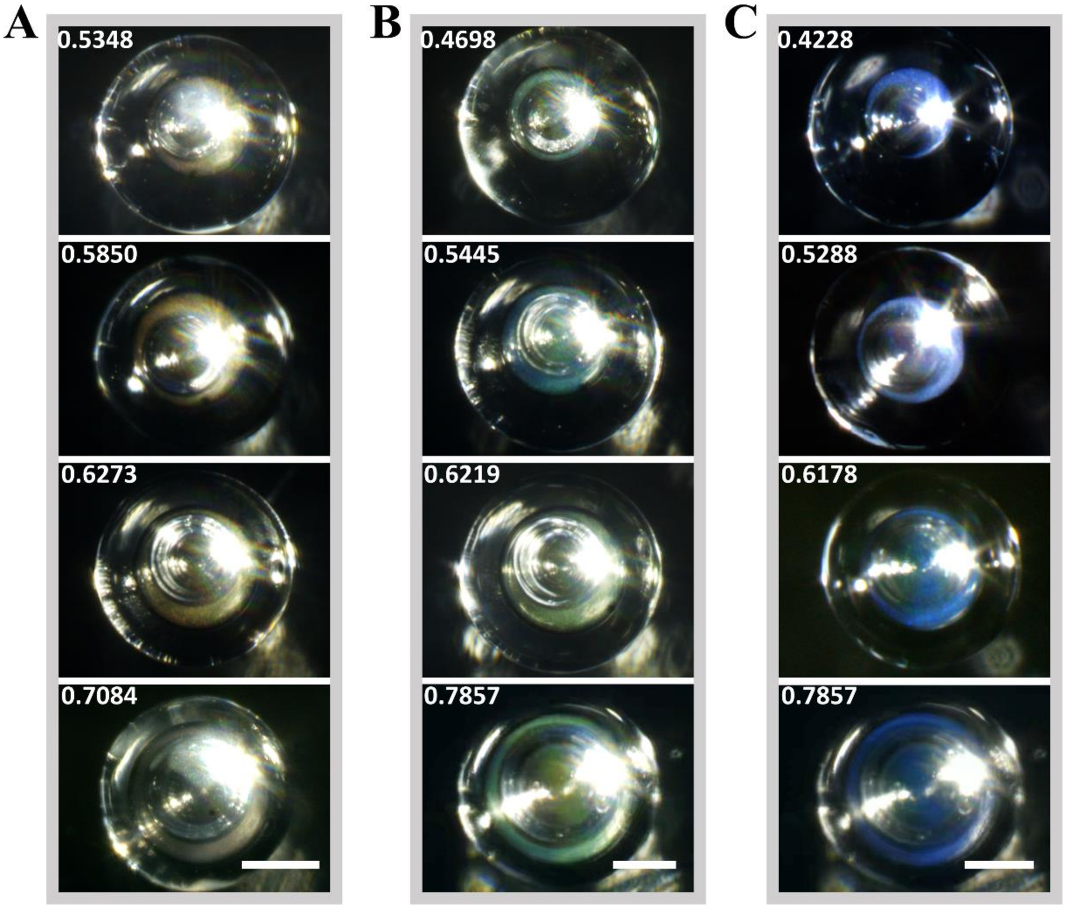

Figure S6. Microscopic images of R, G, B HPC CLCs microbubbles with different r/R. The scale bars are 200μm.

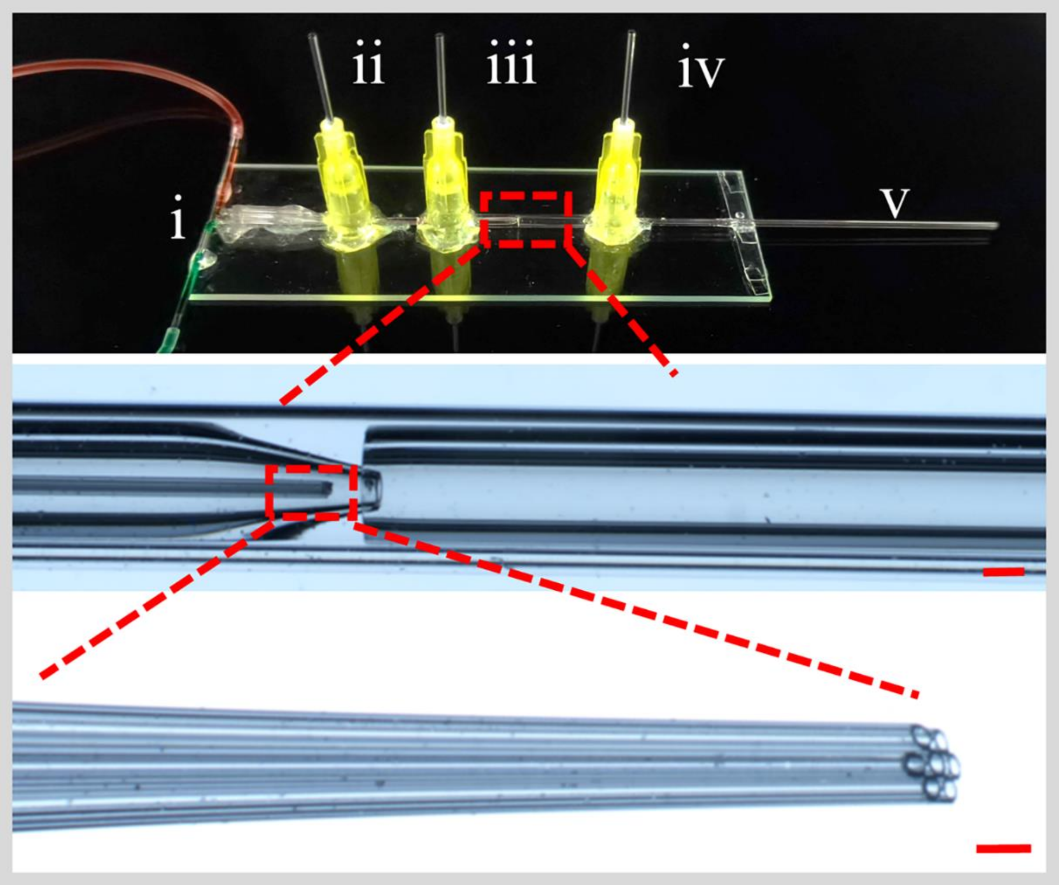

Figure S7. (Top) A photograph of the microfluidic chip with a seven-bore capillary array injection tube. i, ii, iii, iv, v is the inner phase injection tubes, middle phase injection inlet, vent outlet, outer phase injection inlet, and collection tube, respectively. (Middle) a microscopic image showing the coaxially aligned geometry of the tubes. (Bottom) a microscopic image showing the seven-bore injection tube. Scale bars are 200μm (Middle) and 100μm (Bottom), respectively.

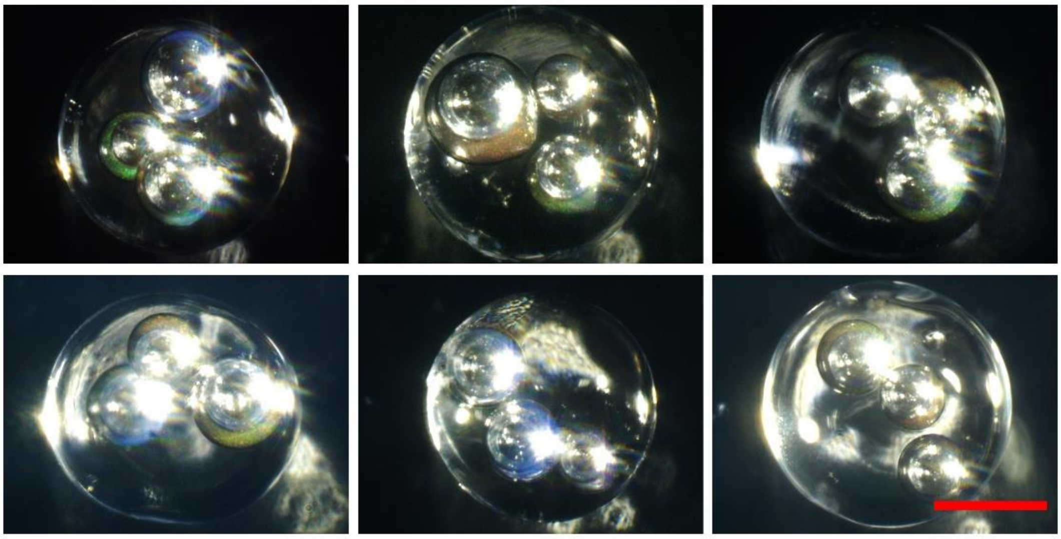

Figure S8. Microscopic images of the three-core HPC CLCs microbubbles with different colors and color combinations. The scale bar is 200μm.

2. Supporting Movie

Movie S1. Sensing state of a yellow-green microbubble under squeezing.
